# Differential Intrinsic Coupling Modes in Psychological and Physical Trauma

**DOI:** 10.3389/fpsyt.2015.00140

**Published:** 2015-10-06

**Authors:** Benjamin T. Dunkley

**Affiliations:** ^1^Department of Diagnostic Imaging, The Hospital for Sick Children, Toronto, ON, Canada; ^2^Neuroscience and Mental Health Program, The Hospital for Sick Children Research Institute, Toronto, ON, Canada; ^3^Department of Medical Imaging, University of Toronto, Toronto, ON, Canada

**Keywords:** post-traumatic stress disorder, mild traumatic brain injury, magnetoencephalography, functional connectivity, resting state

## Introduction

Despite each disorder having a distinct etiology, post-traumatic stress disorder (PTSD), and mild traumatic brain injury (mTBI) often exhibit overlapping symptomatology that makes clinical diagnosis difficult. Furthermore, identification using structural imaging is impractical because anatomical alterations, if they exist at all, can be subtle, or lie beyond the resolution of current technology. Functional MRI, which relies on an indirect measure of brain function (that of blood hemodynamics), has revealed that aberrant functional connectivity (FC) is prevalent in these disorders (1, 2), and machine learning/pattern classification shows promise that these injuries can be objectively classified on certain feature parameters within the spontaneous fluctuations of these signals (3). Although these analyses could potentially aid diagnosis, theories of how these disorders impact underlying *neurophysiological* interactions and neural network function remain scant.

Generating the questions driving answers to this knowledge gap, basic neuroscience research is increasingly revealing the critical role that neurophysiological networks and their dynamics play in cognition and behavior (4). From a philosophical viewpoint, there has been a Kuhnian epistemological paradigm shift from a reductionist, and historically segregative approach toward, or at least combined with, an integrative neural doctrine (5). In other words, ontological standpoints are being driven by neuroscience moving toward the view that mental states are the population-level interactions of neurons, rather than simply the activity of “independent” neurophysiological units; the emergent properties of these networks ultimately give rise to our inner mental life. Understanding how perturbations to these networks results in psychiatric and neurological disorders will be crucial in future explanations, and ultimately the efficacy of diagnostics, intervention, and prognostication.

In this short opinion piece, I discuss some of the Taylor and Pang laboratory’s recent exploratory studies using resting-state paradigms with magnetoencephalography (MEG) that have investigated FC and spontaneous networks in two groups of these patients by examining “intrinsic coupling modes” [ICMs (6)]. These putative types of network interactions comprise distinct mechanisms that facilitate the spatiotemporal organization of ongoing and spontaneous brain activity that defines our psychological state (these modes also subserve goal-directed action, but this is outside the scope of the current piece). I will describe the phenomenology of discrete neurophysiological connectivity profiles evident in the specific cohorts we tested with these disorders, and how they differ in subtle but important ways as well as theorize on the underlying alterations to connectivity that drive these macroscopic markers of disease.

## Coincidence of Symptomatology in Psychological and Physical Trauma

**Figure 1:**
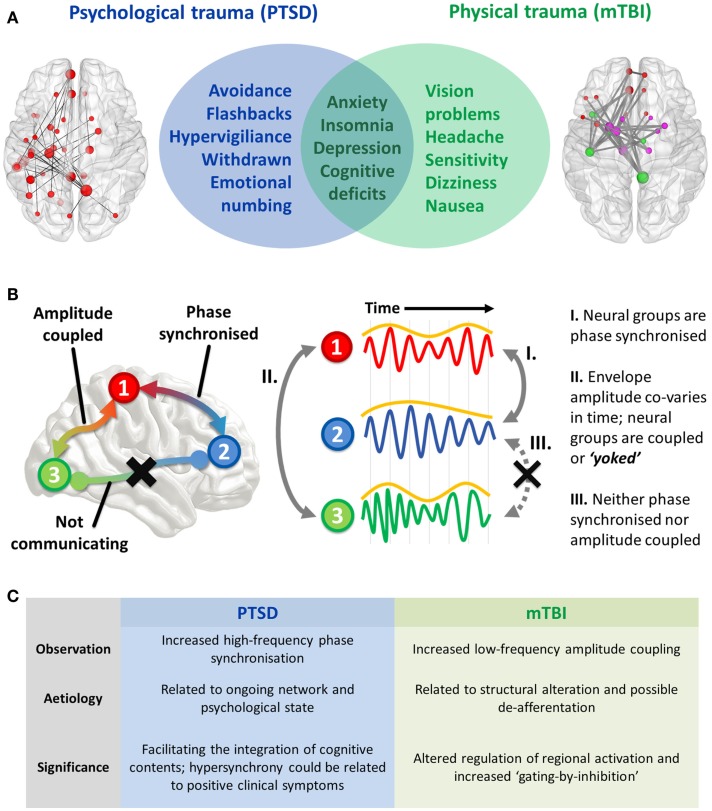
**(A)** Interface of PTSD and mTBI symptomatology, and empirical evidence of altered spontaneous functional connectivity patterns in a resting-state paradigm. Both patient groups show elevated connectivity compared to their respective control groups, with increased coupling in PTSD mediated by *high-frequency* (high gamma-range, 80–150 Hz) oscillatory synchronization; in the mTBI group connectivity is enhanced in the *low-frequency* range (delta–theta range, 1–3 and 3–7 Hz), and is typified by envelope amplitude cross-correlations/temporal covariations. **(B)** Hypothesized role of coupled oscillators in interregional brain communication, and the distinct mechanisms of “intrinsic coupling modes.” These are divided into phase ICMs (facilitating communication between regions 1 and 2, described in I), and envelope ICMs (regulating temporally coordinated activity between regions 2 and 3, described in II). In contrast to these mechanisms, regions 1 and 3 are neither phase synchronized nor amplitude coupled, and therefore communication is suppressed between these regions. **(C)** Summary of findings and the theorized phenomenological significance of these atypical connectivity patterns.

Psychological and physical trauma can give rise to severe psychiatric (PTSD) and neurological (mTBI) disorders that severely affect a patient’s quality of life and impart a huge burden on a healthcare system. These disorders are defined by symptomatology that is often distinct, but in some cases, at the interface between these conditions, overlapping features occur that makes diagnosis difficult for clinicians. A non-exhaustive list of coincident symptoms includes anxiety, depression, cognitive deficits (including attention, memory, and cognitive control), irritability, and insomnia (Figure 1A). Compounding the difficulty of this differentiation is that physical trauma can often lead to PTSD, or mTBI, or a combination of the two; this is especially prevalent in the military. Correct diagnosis is important due to differences in treatment required for these disorders, and this is where delineation based on profiles of brain FC is starting to show promise in both health science and objective assessment. Before the findings are described, I will first explain the types of ICMs [for an in-depth review, see Ref. (6)].

## Mechanisms of Interregional Communication

The two varieties of ICMs that facilitate interregional communication are subserved by (1) the phase synchronization of band-limited oscillations (“phase ICMs”; Figure 1B, I) and (2) the coupling of band-limited amplitude (or power) envelopes (“envelope ICMs”; Figure 1B, II). Phase ICMs can be measured in a variety of ways, but tend to be defined by the degree of coherence/imaginary coherence or synchronization (phase relationship) of the ongoing oscillation between neural populations. Envelope ICMs are computed by the cross-correlation (temporal covariation) of the amplitude (or power) of the oscillatory envelope.

In terms of their functional significance, phase ICMs are thought to support the integration or inhibition of spiking information between two regions. Termed the “communication-through-coherence” hypothesis (7), these “rhythmic fluctuations in excitability” essentially open and close temporal windows of communication that modulate the probability of synaptic input and/or outgoing spiking activity; in other words, depending on the ongoing phase of the oscillation, information is likely to be more readily integrated or suppressed. For example, it might be such that when the phases align, communication between regions is supported, and conversely, when the phases do not align (in anti-phase), communication is inhibited. Therefore, these oscillations provide a way for the brain to dynamically coordinate information in and around the largely static structure (in mesoscopic time scales) of the brain’s circuitry (8). In this way, phase ICMs are highly state dependent, and characterize the ebb and flow of cognitive contents across regions.

The functional role of envelope ICMs is less certain, but they are thought to reflect the coactivation of regions, as well as being more dependent on the underlying structure of neural pathways than phase ICMs. Evidence has shown that types of interactions are also more closely associated with blood-oxygen-level-dependent (BOLD) activity fluctuations (9), and perhaps modulate perceptual and cognitive processing on aperiodic or slow time scales, and could be envisaged as the “yoking” of regions required for a task (or conversely, the inhibition of irrelevant regions). In other words, envelope ICMs appear to represent the coherent fluctuations of coordinated local/regional activity.

So in terms of their general significance, phase ICMs facilitate the integration of information between separate regions across the cortex, while envelope ICMs are thought to regulate the activation (both excitation or inhibition) of relevant or irrelevant regions required for cognition and action. Below, I discuss how these mechanistic properties of neural networks are perturbed in these traumatized populations, and how they differ between disorders.

## Effect of Trauma on Network Dynamics

As part of a larger study examining the utility of MEG for the objective assessment of PTSD in the Canadian Armed Forces, we recruited a cohort of soldiers with combat-related PTSD and a trauma-exposed control group [for details, see Ref. (10)]. For our mTBI group, we recruited patients with an incidence of concussion in the previous 3 months as well as a control group of healthy participants without a history of head injury (11). All groups were subjected to a short battery of cognitive-behavioral tests (measuring disorders of attention, depression, and anxiety), as well as a number of MEG data acquisitions, including both resting state and task. Here, I describe the resting-state data.

We observed increased connectivity in both groups, but importantly, the difference with respect to controls was demarcated by the type of ICM. The PTSD group was distinguished by fast-wave *phase synchronization*, while the mTBI group was found to differ from controls in slow-wave *amplitude coupling* (Figure 1C).

Hyperconnectivity in the PTSD group involved the left hippocampus, temporal, and frontal regions, and I speculate that this reflects some of the primary positive clinical symptoms of PTSD, which are known include disturbing mental imagery and hyperarousal (in contrast to this, there are also negative clinical symptoms in PTSD, including withdrawal, dissociation, and emotional numbing). This is supported by the finding that the left hippocampal connectivity strength to other nodes in the aberrant network was significantly correlated with PTSD symptom severity (measured using the PTSD Check List; future studies will examine how these network measures specifically relate to subscales on the PCL). Previous evidence from intracranial recording supports this position, with studies showing that that hippocampal–cortical gamma synchrony is associated with the formation of episodic memories (12) and a state of vigilance (13). Furthermore, other groups have observed hypersynchronized networks in the disorder, and speculate that this might be due to re-experiencing of traumatic memories (14). Support from resting-EEG studies has also shown that gamma activity may be associated with a state of hyperarousal/vigilance in PTSD (15).

The elevated connectivity seen in the mTBI group was confined to slow-waves (delta, theta, and alpha bands), which at the local level are thought to represent “gating-by-inhibition” (16), and I propose that these regional fluctuations of oscillatory amplitude are reflective of the yoking between areas. This unwanted coupling or yoking, is, in turn, perhaps one of the underlying reasons for a major complaint of mTBI patients, that of a difficulty in mental flexibility and attentional control; anecdotally often referred to as “feeling in a fog.” This position is supported by the finding that large-scale networks mediated by low-frequency amplitude coupling was highly correlated with the inattentive subscale on the Generalized Anxiety Disorder test score; in other words, the degree of covariation in amplitude between two regions was positively associated with attentional problems.

Consequently, I propose that the phenomenological role of these oscillations in these traumatized cohorts is twofold; given previous research on the cortico-hippocampal the high-frequency phase synchronization, I speculate that these interactions seen in our combat-related PTSD group is related to the psychological state of re-experiencing traumatic memories and the other positive clinical symptoms (hypervigiliance, for example), while amplitude covariations in the mTBI group reflects microscopic structural alterations and the unnecessary coupling of brain regions that results in the associated attentional sequelae that typify the disorder. These hypotheses will require testing in future studies by examining how specific symptom clusters of these disorders relate to these connectivity profiles (Figure 1C).

There is a paucity of research examining the relations between these two modes of network action. However, it appears that these mediating mechanisms can function independently of one another (6). In terms of the underlying role of these similar, but distinct, mechanisms, envelope ICMs are theorized to be highly dependent on the underlying structural connectivity (17, 18), with a low-to-moderate dependence on the current cognitive state, and functionally, to be a regulatory mechanism for the temporal coordination of activation in brain regions. Conversely, phase ICMs have a less concrete relation to the physical structure of the network (4, 19), and are crucial in the organization of functional brain networks and their alterations (20). As an extension of this, phase ICMs display a high cognitive state dependence, and reiterating an earlier point, as regulatory in the integration (or suppression) of information across brain regions (6, 10).

## Conclusion

In summary, we observed elevated MEG connectivity in both patient groups that was differentiated by the frequency and mechanism of coupling. In terms of the group differences we found in envelope ICM elevations in mTBI, I propose that these alterations are closely related to structural changes, such as white matter damage and deafferentation that subsequently impart secondary consequences on cognition. In PTSD, the hypersynchrony of this group could a marker of re-experiencing of disturbing mental imagery and hyper-vigilant states. Furthermore, we found that the alterations in connectivity evident in patient groups appear to explain some of the cognitive sequelae that typify the symptoms of these disorders. Despite this, we should exercise caution in attribution of any specific functional roles to canonical oscillations, as emerging evidence suggests the role of these “spectral fingerprints” are far more complicated than our current understanding, and that the underlying neural processes that mediate brain/mental states cannot simply be ascribed to one frequency, but the interplay within and among them. Moving forward, there is potential to use these macroscopic functional markers in a diagnostic, prognostic, and intervention fashion, and particularly in guiding future interventionist treatments, such as rTMS and TDCS, that appear to modulate the intrinsic oscillatory state of the brain and its topological nature.

## Conflict of Interest Statement

The author declares that the research was conducted in the absence of any commercial or financial relationships that could be construed as a potential conflict of interest.
